# Growth, Heavy Metal Accumulation, and Translocation in Chamomile (*Matricaria recutita* L.) and Safflower (*Carthamus tinctorius* L.) Under Severe Cd and Pb Contamination in an Alkaline Soil

**DOI:** 10.3390/plants15203088

**Published:** 2026-10-09

**Authors:** Christos Kikis, Aspasia Grammenou, Georgios Thalassinos, Spyridon A. Petropoulos, Vasileios Antoniadis

**Affiliations:** Department of Agriculture, Crop Production and Rural Environment, University of Thessaly, 38446 Volos, Greece; chkikis@uth.gr (C.K.); asgrammenou@uth.gr (A.G.); gthalassinos@uth.gr (G.T.); antoniadis@uth.gr (V.A.)

**Keywords:** potentially toxic elements, growth stimulation, metal accumulation, phytoremediation, metal availability, phytomanagement

## Abstract

Severe cadmium (Cd) and lead (Pb) contamination can compromise the productive and safe use of agricultural and degraded soils, yet plant tolerance, metal partitioning, and removal efficiency under alkaline soil conditions remain insufficiently resolved. This study aimed to evaluate growth responses, Cd and Pb accumulation, root-to-shoot translocation, and phytoremediation traits in chamomile (*Matricaria recutita* L.) and safflower (*Carthamus tinctorius* L.) under severe Cd and Pb contamination in an alkaline soil. A 4-month greenhouse experiment used 120 independent 5-L pots containing alkaline soil amended with 0, 50, or 100 mg Cd kg^−1^ and 0, 1500, or 3000 mg Pb kg^−1^. Chamomile accumulated 138.6 mg Cd kg^−1^ DW in shoots at Cd50, with a Bioconcentration Factor (BCF) value of 3.04 and a Translocation Factor (TF) of 1.56, whereas 100 mg Cd kg^−1^ caused severe biomass suppression and reduced translocation. Safflower showed significant growth stimulation at Pb1500, with shoot dry and fresh weight increasing by 17% and 44%, respectively, while Pb remained predominantly belowground (TF < 1); growth was strongly inhibited at Pb3000. Aboveground removal remained low, representing approximately 0.18% of the measured post-harvest soil Cd pool for chamomile at Cd50 and 0.008% of the soil Pb pool for safflower at Pb1500. Chamomile therefore warrants repeated-harvest evaluation for Cd phytoextraction, whereas safflower warrants long-term assessment for Pb phytostabilization-oriented management. Field and multi-season studies are needed to verify remediation efficiency, metal mobility, and safe biomass management.

## 1. Introduction

Soil contamination by potentially toxic elements (PTEs) represents a critical driver of soil functional decline, directly affecting food safety and thus human well-being [1]. Elevated concentrations of highly toxic PTEs like cadmium (Cd) and lead (Pb) persist in soils throughout the world. At the plant level, Cd and Pb can impair growth through disruption of nutrient uptake and ion homeostasis, inhibition of root development and photosynthetic activity, and induction of oxidative stress [2]. Cd is generally characterized by relatively high mobility in the soil–plant system and can be efficiently taken up by roots and transferred to aboveground tissues, where it may interfere with cellular metabolism and biomass production [3]. Pb is typically less mobile within plants and is often retained predominantly in roots; nevertheless, elevated Pb exposure can induce oxidative damage, impair physiological and metabolic processes, and strongly suppress plant growth [4]. These effects ultimately reduce plant establishment and productivity and may increase the risk of contaminant transfer into agricultural or economically valuable biomass.

Alkaline soils present a particular challenge for the management of PTE contamination because high pH and carbonate content strongly influence metal sorption, speciation, mobility, and plant availability [5,6]. Nevertheless, anthropogenic activities can increase soil Cd and Pb concentrations far beyond natural background levels, particularly in mining areas, industrial hotspots, and contaminated agricultural lands. Phytoremediation is a plant-based approach for the management of contaminated soils that exploits processes such as metal uptake, translocation, and stabilization while minimizing physical disturbance of the soil matrix [7]. Compared with conventional excavation or intensive physicochemical remediation, it can offer a less disruptive and potentially more sustainable option for large or moderately contaminated areas where complete soil removal may be technically or economically impractical [7]. In mining-affected, industrial, brownfield, and other degraded sites, vegetation can also contribute to soil cover, erosion control, and the reduction in contaminant dispersion, while selected plant species may promote either phytoextraction through accumulation in harvestable shoots or phytostabilization through retention of contaminants in the root zone [8]. However, the effectiveness of these approaches depends not only on metal concentration in plant tissues, but also on plant tolerance, biomass production, metal availability, translocation patterns, and the capacity to maintain these responses over repeated growth cycles [9]. Therefore, identifying plant species that can remain productive while accumulating or retaining PTEs under severe contamination is an important step toward the development of realistic phytoremediation and phytomanagement strategies. 

*Matricaria recutita* L. (chamomile) is a widely used medicinal plant and a species of interest for Cd phytoremediation. Chamomile is important for human use because its flowers and extracts are extensively consumed in herbal teas, traditional medicines, cosmetics, and pharmaceutical preparations, and it has documented anti-inflammatory, antimicrobial and wound healing applications [10]. From a phytoremediation perspective, previous studies have reported contrasting Cd accumulation and partitioning patterns in chamomile. Several studies found substantial Cd accumulation in shoots, flowers, or other aboveground tissues, with good translocation to harvestable organs [11,12,13]. In contrast, other studies reported Cd retention in roots and lower shoot-to-root transfer, indicating that chamomile behaves more as a tolerant non-accumulator under some exposure conditions [14,15]. Evidence also shows that this variability depends strongly on growth conditions, substrate, and soil properties: aerial Cd was higher under greenhouse conditions whereas roots retained more Cd under field conditions, Cd uptake differed across soils, and soil pH and cation exchange properties are recognized regulators of metal mobility and plant availability [16]. This point is especially important because chamomile products intended for human consumption can become unsafe when grown on contaminated soils, as Cd can accumulate in flowers and infusions above allowable levels even at environmentally relevant contamination [17].

*Carthamus tinctorius* L. (safflower) combines agricultural and medicinal value with notable tolerance to PTE-stress, particularly Pb, making it a promising species for phytomanagement of contaminated soils [18]. Safflower is important for humans because it is cultivated for high-quality edible oil, is rich in linoleic acid, provides dyes and food colorants, and has long-standing medicinal uses in traditional and modern systems for human health [19]. Under Pb stress, safflower shows physiological resilience linked to antioxidant and metabolic defense responses. Pb exposure induces oxidative stress and biomass reduction, but safflower can maintain survival through the activation of defense systems, including increased glutathione, glyoxalase activity, and phytochelatin-related detoxification pathways [20]. Genetic evidence also supports inherent Pb tolerance, as the CtWD40-6 pathway positively regulates Pb tolerance and accumulation in safflower and enhances its remediation capacity [21]. At the same time, safflower often retains more Pb in roots than leaves, which helps explain its tolerance but can limit efficient Pb extraction from soil [18]. This makes safflower especially relevant for phytomanagement strategies in which contaminated land is kept productive through non-food or industrial uses.

Although Cd and Pb responses have previously been investigated in chamomile and safflower, respectively, their performance under more severe contamination remains less well resolved, particularly in alkaline calcareous soils. In chamomile, soil studies using up to 30 mg Cd kg^−1^ reported increasing Cd accumulation accompanied by reductions in growth [22], while hydroponic experiments showed preferential Cd retention in roots, where concentrations were approximately 7–11 times higher than in leaves under 60–120 μΜ Cd exposure [14]. In safflower, a study conducted in slightly alkaline soil (pH 7.4) showed that increasing Pb contamination from 450 to 900 mg kg^−1^ reduced the aboveground bioconcentration factor (BCF) from 0.12 to 0.07 and the translocation factor (TF) from 0.79 to 0.61 [18]. Likewise, at 1200 mg Pb kg^−1^, shoot and root dry biomass declined by 46.9 and 53.2%, respectively, while Pb translocation remained very low (TF < 0.22) [23]. Collectively, these findings demonstrate an important trade-off: increasing metal exposure and tissue accumulation may be accompanied by biomass suppression and restricted root-to-shoot transfer, thereby limiting effective aboveground metal removal. Consequently, the relationships among soil metal bioavailability, plant growth, tissue accumulation, translocation, and biomass-dependent metal uptake require further evaluation at severe contamination levels under alkaline conditions. Such an integrated assessment is necessary to distinguish effective phytoextraction from predominantly root-based metal retention and phytostabilization.

Thus, this study aimed to provide a targeted proof-of-concept screening to (1) evaluate whether safflower and chamomile can withstand high, hotspot-level concentrations of Cd and Pb; (2) characterize Cd accumulation and root-to-shoot translocation in chamomile under severe Cd exposure in alkaline soil; and (3) characterize safflower growth and Pb partitioning responses across severe Pb contamination levels in an alkaline soil environment. By linking plant tolerance and biomass production with metal uptake, the study provides evidence for assessing whether these species exhibit traits more suitable for metal removal or stabilization in the root zone. From a practical perspective, this information can assist researchers and land managers in selecting plant-based approaches for severely contaminated soils, while helping to identify situations in which metal accumulation may restrict the safe agricultural use of the produced biomass. For farmers and other land users managing contaminated or marginal land, such information is relevant for evaluating whether cultivation can provide a productive function without overlooking contamination-related risks. More broadly, the findings provide a scientific basis for further field validation of plant-based strategies aimed at limiting the mobility and environmental transfer of Cd and Pb and supporting the safer management of contaminated land.

## 2. Materials and Methods

### 2.1. Soil Sampling

Baseline non-contaminated alkaline agricultural soil was sampled (0–30 cm depth) at Velestino, Magnesia, Greece (39.39541° N, 22.75746° E). Soil subsamples were collected from multiple sampling points across the sampling area following the Z-shape procedure. Samples were combined and thoroughly homogenized to obtain a representative composite soil sample for the experiment. The four-month greenhouse pot experiment was conducted from February to May 2024 at the experimental greenhouse of the University of Thessaly, School of Agricultural Sciences (39.38796° N, 22.94016° E). The composite sample was air-dried, passed through a 2-mm sieve, and partitioned into 3-kg plastic bags. Its properties included pH values of 7.9 (H_2_O), 1.1% organic carbon (Walkley–Black method), and 20% calcium carbonate content (CaCO_3_) (Bernard calcimeter; manufacturer and model not available; University laboratory equipment). Particle-size distribution (Bouyoucos ASTM 152H soil hydrometer; manufacturer/model not available, older laboratory equipment) was 45% sand, 39% silt, and 16% clay, classifying the soil as loam [24]. Pseudo-total Cd (0.05 mg kg^−1^) and Pb (3.57 mg kg^−1^) concentrations were well below regulatory thresholds, confirming its non-contaminated status.

### 2.2. Soil Spiking Process

Exposure treatments were established on a dry-soil-mass basis at nominal concentrations of 0, 50, and 100 mg Cd kg^−1^ soil and 0, 1500, and 3000 mg Pb kg^−1^ soil. Each experimental unit initially contained 3.0 kg of dry soil. Accordingly, the Cd50 and Cd100 treatments required the addition of 150 and 300 mg elemental Cd per experimental unit, respectively, whereas the Pb1500 and Pb3000 treatments required 4.50 and 9.00 g elemental Pb per experimental unit, respectively.

Metals were supplied as anhydrous CdCl_2_ and PbCl_2_, respectively. Based on their molecular composition, the corresponding salt additions per 3-kg soil unit were 0.2446 g CdCl_2_ for Cd50, 0.4892 g CdCl_2_ for Cd100, 6.0398 g PbCl_2_ for Pb1500, and 12.0796 g PbCl_2_ for Pb3000. The Cd and Pb concentrations were selected to represent severe to hotspot-level soil contamination rather than typical background levels in agricultural soils. The selected concentrations fall within the order of magnitude reported for heavily impacted mining soils, including Lavrion (Attica, Greece; mean Cd 39.5, max 287.7 mg kg^−1^; [25]), Stratoni (Chalkidiki, Greece; peak 37 mg Cd kg^−1^; [26]), and Tunisia’s Jalta (42 mg kg^−1^; [27]) and Jebel Ressas mines (231 mg Cd kg^−1^; [28]). For Pb, extreme concentrations can be found in Spain—including Cartagena (2633 mg kg^−1^; [29]), La Garza Basin (3050 mg kg^−1^; [30]), and Valle de Alcudia (1481 mg kg^−1^; [31])—and Acerra, Italy (1099 mg Pb kg^−1^; [32]). The Cd and Pb concentrations applied in the present study fall within the range reported for these documented contamination hotspots. For each metal, two increasing contamination levels were selected, with the higher concentration being twice the lower one, to evaluate changes in plant tolerance, metal accumulation, and translocation under increasing metal stress. The different concentration ranges used for Cd and Pb reflect metal-specific experimental concentration ranges and were not intended to represent equivalent toxic doses.

Each treatment comprised 10 experimental units per species. Treatment amendments were therefore prepared at a final volume of 2 L, allowing a 100-mL aliquot to be applied to each 3-kg soil unit. For the Cd treatments, 4.8924 and 9.7845 g anhydrous CdCl_2_ were used for Cd50 and Cd100, respectively, corresponding to approximately 3.00 and 6.00 g elemental Cd in each 2-L preparation. For the Pb treatments, 120.8 and 241.6 g anhydrous PbCl_2_ were used for Pb1500 and Pb3000, respectively, corresponding to approximately 90.0 and 180.0 g elemental Pb in each 2-L preparation. The salts were added to deionized water under continuous stirring until the solution appeared visually clear. Each 100-mL aliquot therefore provided approximately 150 or 300 mg Cd to the Cd50 and Cd100 treatments, respectively, and 4.50 or 9.00 g Pb to the Pb1500 and Pb3000 treatments, respectively. Control soils received an equivalent volume (100 mL) of deionized water without Cd or Pb added.

Because the metals were applied as chloride salts, the treatments also introduced accompanying chloride. Based on the stoichiometric chloride content of CdCl_2_ and PbCl_2_, the corresponding chloride additions were approximately 0.0315 and 0.0631 g Cl kg^−1^ soil for Cd50 and Cd100, respectively, and 0.513 and 1.027 g Cl kg^−1^ soil for Pb1500 and Pb3000, respectively. Cd and Pb were applied as chloride salts to provide defined inorganic metal sources while avoiding the introduction of nutritionally relevant counter-ions that could independently modify plant responses. For example, nitrate salts (most common nutritionally derived metal salts) were avoided because they can introduce treatment-dependent amounts of plant-available N, which can influence plant growth, metal tolerance, and Cd accumulation and translocation under metal stress [33]. Chloride salts are widely applicable for controlled metal-spiking experiments and provide an initially reactive metal input; however, chloride may itself influence metal speciation and mobility, particularly through chlorocomplex formation [34]. Therefore, a chloride-only control was prospectively included in the original experimental design and was conducted concurrently with the main treatments to evaluate whether the chloride introduced with PbCl_2_ could independently influence plant growth. The chloride-control treatment was prepared using CaCl_2_·2H_2_O at a chloride loading equivalent to that introduced by Pb3000. The Pb3000 treatment supplied approximately 1.027 g Cl kg^−1^ dry soil, corresponding to approximately 3.08 g Cl per 3-kg soil unit. This chloride dose corresponds to approximately 6.39 g CaCl_2_·2H_2_O per experimental unit. The chloride-control pots were subjected to the same incubation, greenhouse conditions, randomization, pot repositioning, irrigation, and harvest procedures as the main treatments. As this treatment served specifically to assess a potential chloride-associated growth effect, its results are presented separately in the Supplementary Material (Figure S1).

Following amendment, soils were thoroughly mixed every two days and incubated for 30 days at 25 ± 2 °C in the dark. Soil moisture was maintained gravimetrically at 65% field capacity. Nominal metal concentrations were calculated relative to the initial 3.0-kg dry soil mass; the perlite subsequently added during pot establishment was not included in the calculation of nominal contamination levels.

### 2.3. Experimental Design

Following incubation, the soils were transferred into standard 5-L pots, each containing 3 kg of dry soil and 1 kg of perlite (3:1 ratio) to improve drainage, aeration, and substrate uniformity under metal stress. Certified seeds of *Matricaria recutita* L. and *Carthamus tinctorius* L. (Pharmasaat GmbH, Artern, Germany) were sown at a rate of three seeds per pot. The experimental design comprised three components: a Cd exposure series, a Pb exposure series, and a concurrently established chloride-only control. The Cd exposure series included 50 and 100 mg Cd kg^−1^ soil (Cd50 and Cd100), whereas the Pb exposure series included 1500 and 3000 mg Pb kg^−1^ soil (Pb1500 and Pb3000), with 10 replicate pots established for each species × treatment combination. A common untreated treatment (0 mg kg^−1^) served as the reference control for both metal exposure series. In addition, a chloride-only control was established concurrently to account for potential effects associated with the chloride salts. Overall, the experiment comprised 120 pots, corresponding to 10 independent experimental units for each species × treatment combination, with each pot representing one experimental unit. The entire pot experiment occupied approximately 5 m^2^ of greenhouse area. The 4-month greenhouse experiment was conducted under natural light (25 ± 3 °C and 40% relative humidity). The greenhouse environment was monitored and confirmed to be spatially uniform in terms of light exposure, temperature, and humidity across the growing area, precluding the need for formal spatial blocking. Pots were nonetheless arranged according to a complete randomized design, with initial pot positions assigned using a random number generator and were systematically rotated within the greenhouse every 7 days throughout the 4-month experimental period to further eliminate any residual, unmeasured positional effects related to light availability, temperature variation, or irrigation distribution. Biomass and analytical measurements were performed using standardized, instrument-based procedures (calibrated balances and inductively coupled plasma mass spectrometry (ICP-MS) quantification against certified reference standards—see below); as these measurements are objective and not subject to observer judgment, formal blinding of personnel to treatment identity during measurement was not implemented, consistent with common practice for instrument-based endpoints in plant physiology and soil science studies. An overall illustration of the experimental layout is shown in Figure S2 in the supplemental material.

### 2.4. Soil and Plant Analysis

At harvest, aboveground biomass was cut 2 cm above the soil surface and shoot fresh weight (FW) was immediately weighed. Roots were separated from soil, rinsed under running tap water to remove bulk adhering soil particles, immersed in 10 mM ethylenediaminetetraacetic acid (EDTA) for 15 min at room temperature (24 °C) to desorb surface-bound, non-internalized metal fractions, and then rinsed thoroughly with deionized water. Roots were visually inspected to confirm the absence of residual soil particles prior to drying. Plant samples were stored in pre-weighed paper bags, oven-dried at 70 °C (24–48 h) to constant dry biomass, milled, and stored in plastic jars. For Cd and Pb analysis, 1 g of powder underwent dry ashing at 450 °C for 5 h in a muffle furnace; this temperature avoids Cd volatilization, ensuring high recoveries (91.6–108%) without metal loss [35]. The ash was extracted with 10 mL of 20% HCl [36].

For soil analysis, three homogenized cores per pot formed a single composite sample. After harvest, soil was air-dried, sieved (2-mm), and put in plastic jars. Analyses included pH (1:2.5 H_2_O) and organic carbon (Walkley–Black wet oxidation) evaluation. Pseudo-total Cd and Pb were determined via aqua regia digestion (concentrated HCl:HNO_3_, 3:1 ratio) for 5 h at 140 °C [37]. Available Cd and Pb fractions were extracted using the diethylenetriaminepentaacetic acid (DTPA)–TEA–CaCl_2_ method (0.005 M DTPA, 0.01 M CaCl_2_ and 0.1 M triethanolamine), adjusted to pH 7.3, following the method of Rowell [38]. These two extraction schemes target conceptually distinct soil metal pools: pseudo-total concentrations (aqua regia digestion) reflect the near-complete elemental content of the soil, including both mobile and strongly bound, non-labile fractions, whereas DTPA-extractable concentrations reflect a more labile, chelator-accessible pool considered representative of the plant-available fraction. The buffered DTPA–TEA–CaCl_2_ method was specifically selected because it is well established as an appropriate bioavailability proxy in calcareous, alkaline soils, where high pH and carbonate content strongly influence metal speciation and can bias simpler, unbuffered extractants [39]. Suspensions were centrifuged at 3500 rpm for 15 min and filtered [40]. All extract analytes were quantified via ICP-MS (Thermo iCAP TQ, Bremen, Germany).

### 2.5. Indices for the Estimation of the Phytoremediation Potential

#### 2.5.1. Bioconcentration Factor

The bioconcentration factor (BCF) was used to describe the relationship between metal concentration in plant tissues and the corresponding pseudo-total soil metal concentration [41]. As the most common indicator in phytoremediation, BCF assesses substrate-to-tissue pollutant absorption, enabling comparisons of extraction and accumulation capacities among species. The BCF is calculated by dividing the PTE content (mg kg^−1^) in plant tissues by the pseudo-total soil PTE content (mg kg^−1^) [42]. Thus, the formula BCF is BCF = Cplant/Csoil, where Cplant is the concentration of the metal in plant tissues (mg kg^−1^), and Csoil is the pseudo-total concentration of the metal in soil (mg kg^−1^).

#### 2.5.2. Translocation Factor

The translocation factor (TF) indicates root-to-aboveground PTE translocation within plant tissues. TF values > 1 show prioritized PTE movement and storage in aerial parts, indicating phytoextraction ability. TF is calculated by dividing aboveground biomass PTE concentration (mg kg^−1^) by root PTE concentration (mg kg^−1^) [43]. Thus, TF = Cshoot/Croot, where Cshoot and Croot represent metal concentrations (mg kg^−1^) in shoots and roots, respectively. For each treatment, BCFshoot, BCFroot, and TF were calculated separately for each experimental unit using the corresponding pot-level soil and tissue concentrations. Treatment values reported in the text and figures therefore represent the mean ± SD of these replicate-level indices and were not obtained by dividing treatment-mean tissue concentrations by treatment-mean soil or root concentrations. All plant tissue metal concentrations used in these calculations are expressed on a dry weight (DW) basis, as all plant material was oven-dried to constant weight prior to metal analysis.

For clarity, key terms are used as follows throughout this manuscript: uptake refers to the total mass of metal absorbed by plant tissue (expressed as µg per pot), whereas accumulation refers to the metal concentration within a given tissue (expressed as mg kg^−1^ DW). A TF > 1 is interpreted here as evidence of preferential root-to-shoot translocation, a trait relevant to phytoextraction, whereas a TF < 1 indicates preferential belowground retention, a trait compatible with phytostabilization-oriented phytomanagement. These indices are used as screening indicators and are not considered, by themselves, as evidence of effective field-scale phytoextraction or long-term phytostabilization.

### 2.6. Quality Control and Statistical Analysis

Analytical quality control was ensured via triplicate measurements, blank analyses, and precision monitoring (RSD < 10%). Plant analysis accuracy was confirmed through matrix spikes (90–110% recovery). Soil accuracy was assessed using an in-house standard and certified reference material BCR-141R (Calcareous Loam Soil; European Commission, Joint Research Centre, Geel, Belgium), achieving recoveries of 93–108%.

Statistical differences among contamination levels were evaluated using one-way ANOVA at *p* < 0.05. Parametric assumptions were assessed using the Shapiro–Wilk test for normality and Levene’s test for homogeneity of variances. For homoscedastic datasets (Levene’s test, *p* > 0.05), standard one-way analysis of variance (ANOVA) followed by Tukey’s honestly significant difference (HSD) post hoc test was applied. For datasets exhibiting significant heteroscedasticity (Levene’s test, *p* < 0.05), including pseudo-total and DTPA-extractable soil Cd and Pb concentrations, plant tissue metal concentrations, and metal uptake, Welch’s ANOVA followed by the Games–Howell post hoc test was used.

To further explore multivariate relationships among plant growth, soil metal availability, tissue metal accumulation, and shoot metal uptake, principal component analysis (PCA) was performed separately for the Cd and Pb datasets. Each PCA comprised 60 independent observations, corresponding to two plant species × three treatment levels × 10 replicate pots. Eight quantitative variables were included in each analysis: shoot DW, root DW, shoot FW, plant height, DTPA-extractable metal concentration, shoot metal concentration, root metal concentration, and shoot metal uptake. PCA was performed using the correlation matrix to account for differences in measurement units and variable scales. Species × treatment combinations were used as grouping variables for graphical visualization and were not included as active variables in the PCA. All principal components were extracted, whereas the first two components (PC1 and PC2) were used for graphical representation in the biplots.

The study was conducted as a single greenhouse experiment comprising two metal-specific dose series that were run concurrently under the same environmental conditions: a Cd series (control, Cd50, and Cd100) and a Pb series (control, Pb1500, and Pb3000). The same untreated control group served as the reference for both metal-specific series. Because Cd and Pb represent different contaminants applied over different concentration ranges, no direct statistical comparisons were performed between Cd and Pb treatments; instead, the Cd and Pb dose series were analyzed separately. Accordingly, for each species and metal, statistical analysis comprised three treatment levels: control, Cd50, and Cd100 for the Cd series and control, Pb1500, and Pb3000 for the Pb series. Each treatment contained 10 independent experimental units (pots).

The chloride-only treatment was not included in the metal dose–response ANOVAs because it served a separate control function. For each species, the chloride-only treatment was compared with the untreated control after verification of normality and homogeneity of variance to determine whether chloride alone, at the loading corresponding to Pb3000, significantly affected plant growth.

Analyses were performed using IBM SPSS Statistics v.29 (USA), and figures were generated using OriginPro v.10.3. Full statistical outputs, including sample sizes, degrees of freedom, F or Welch statistics, exact *p*-values, and effect sizes (η^2^), are provided in Table S2 of the Supplementary Material.

## 3. Results

### 3.1. Concentrations of Cd and Pb in Soil

Pseudo-total Cd concentrations increased with Cd addition in both plant species, from control levels (0.08 and 0.054 mg kg^−1^ for chamomile and safflower, respectively) to 45.6 and 46.1 mg kg^−1^ at Cd50, and 95.4 and 96.2 mg kg^−1^ at Cd100. Pseudo-total Pb concentrations also increased from 4.47 and 3.37 mg kg^−1^ in control treatments (chamomile and safflower, respectively) to 1226 and 1233 mg kg^−1^ at Pb1500, and 2722 and 2773 mg kg^−1^ at Pb3000. DTPA-extractable Cd and Pb concentrations significantly increased (*p* < 0.001) along the contamination gradient. DTPA-extractable Cd corresponded to 30–36% of total Cd at Cd50 (16.2 and 13.7 mg kg^−1^ for chamomile and safflower, respectively) and exceeded 53% of total Cd at Cd100 (51.1 and 51.8 mg kg^−1^, respectively). Similarly, DTPA-extractable Pb corresponded to 44–47% of total Pb at Pb1500 (542 and 580 mg kg^−1^, for chamomile and safflower, respectively) and 51–53% at Pb3000 (1415 and 1437 mg kg^−1^, respectively). Soil Cd and Pb concentrations are presented in the supplementary material (Table S1), together with supporting information.

### 3.2. Plant Growth Under Cd and Pb Stress

**Figure 1:**
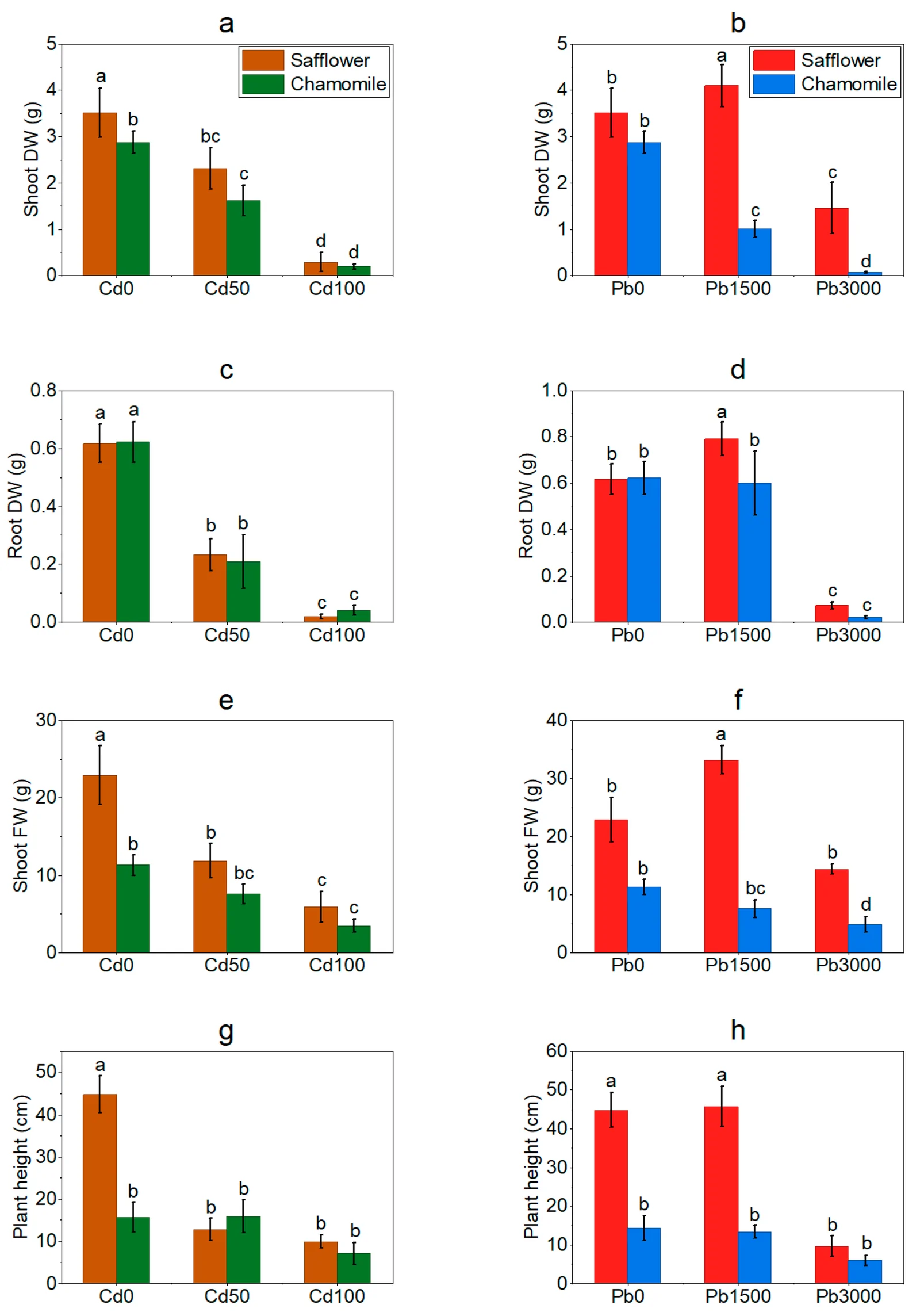
Effect of Cd and Pb stress on growth parameters of safflower and chamomile plants. (**a**,**b**) Shoot dry weight (DW), (**c**,**d**) root DW, (**e**,**f**) shoot fresh weight (FW), and (**g**,**h**) plant height. Left panels (**a**,**c**,**e**,**g**) represent responses to Cd concentrations (0, 50, and 100 mg kg^−1^), whereas right panels (**b**,**d**,**f**,**h**) represent responses to Pb concentrations (0, 1500, and 3000 mg kg^−1^). Data are expressed as mean ± SD (*n* = 10). Statistical differences among contamination levels within each species and metal-specific dose series were evaluated using one-way ANOVA followed by Tukey’s HSD post hoc test (*p* < 0.05). Different lowercase letters indicate significant differences among contamination levels within the corresponding species and metal-specific dose series. No statistical comparisons were performed between Cd and Pb treatments or between the species.

Metal treatments induced distinct biomass responses in the studied species (Figure 1). In safflower, Cd stress caused a severe, dose-dependent growth reduction; Cd50 significantly decreased shoot DW to 2.3 g (−34%, *p* < 0.001) and Cd100 further suppressed it to 0.3 g, compared to the control (3.5 g) (Figure 1a), mirroring the pattern also observed in shoot FW (Figure 1e). Conversely, Pb1500 elicited a pronounced stimulatory effect on biomass, significantly increasing safflower shoot DW to a maximum of 4.1 g (+17%, *p* < 0.001) and FW to 33 g (+44%, *p* < 0.001) compared to the control (22.9 g FW) (Figure 1f). This stimulatory effect was reversed at Pb3000, where shoot DW declined to 1.5 g (Figure 1b). In chamomile, both PTEs induced a steady and significant decline in the aerial biomass with no comparable growth stimulation. Control plants exhibited the highest shoot DW (2.9 g), which consistently decreased to 1.6 g (Cd50), 0.2 g (Cd100), 1.0 g (Pb1500), and near-zero levels at Pb3000 (Figure 1a,b). However, chamomile FW showed no significant differences under Cd50 and Pb1500 compared to the control.

Root biomass strictly reflected the aerial growth trends for both species (Figure 1c,d). In particular, safflower root DW was markedly impaired by Cd, decreasing from 0.62 g (control) to nearly undetectable levels at Cd100. Conversely, Pb1500 significantly promoted root proliferation, peaking at 0.79 g, before dropping to 0.07 g under Pb3000. Chamomile root growth was significantly decreased when exposed to PTEs; compared to the control (0.62 g), root DW was severely restricted by Cd (0.21 g at Cd50; 0.04 g at Cd100) and was almost completely inhibited at Pb3000 (Figure 1c,d).

Safflower height was significantly stunted by high Cd/Pb levels (10–13 cm vs. 44.2 cm for the control treatment), whereas Pb1500 maintained control-level heights (46.8 cm). Chamomile height remained stable (13–14 cm) under moderate Cd and Pb concentrations but declined to 6.0–6.5 cm at the highest PTE levels (Figure 1g,h).

### 3.3. Concentrations of Cd and Pb in Plants

**Figure 2:**
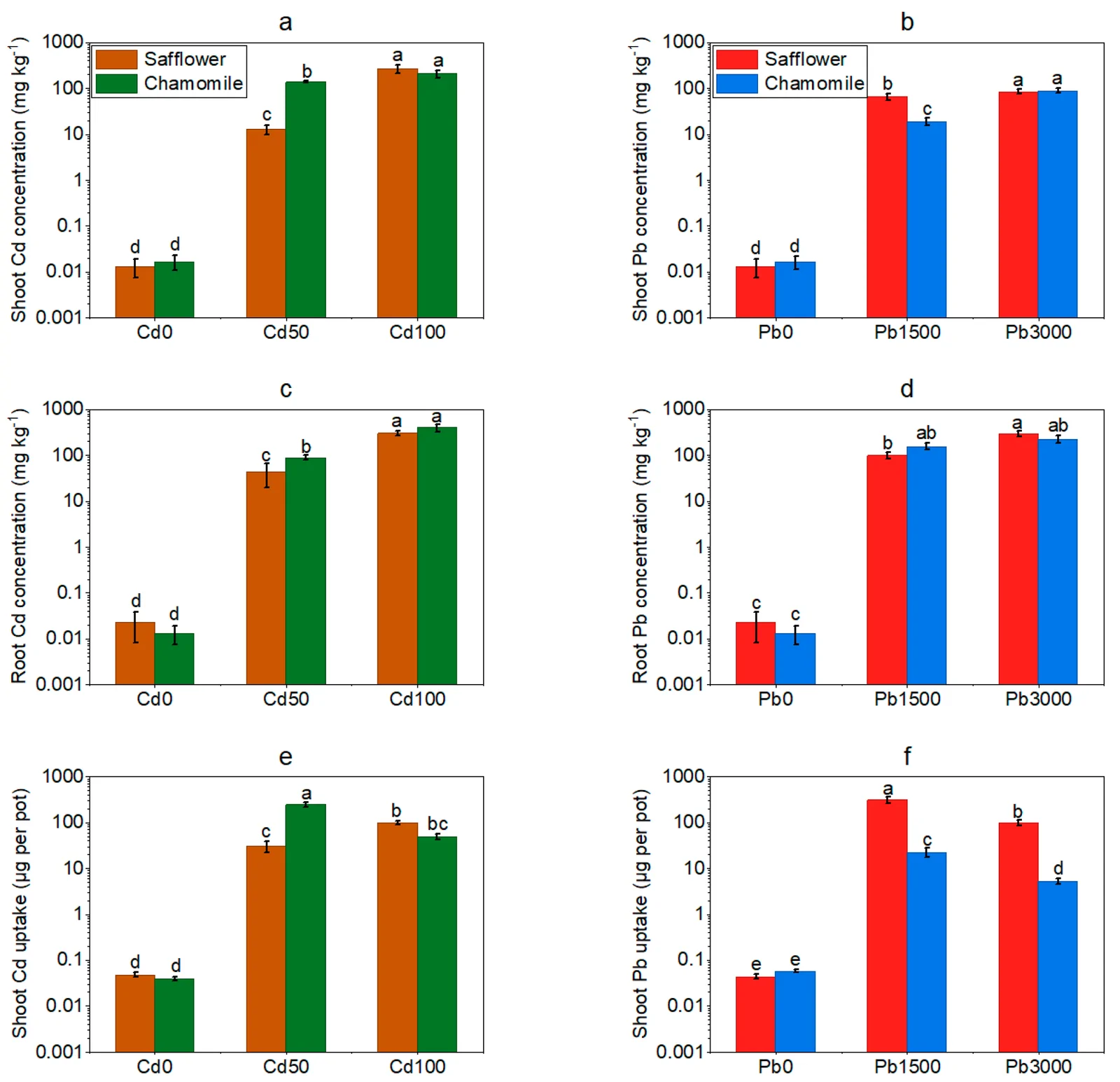
Concentrations (mg kg^−1^) of Cd and Pb in shoots (**a**,**b**) and roots (**c**,**d**), and total shoot uptake (μg per pot) for Cd (**e**) and Pb (**f**). Treatments included control (unamended soil), Cd50 (50 mg Cd kg^−1^), Cd100 (100 mg Cd kg^−1^), Pb1500 (1500 mg Pb kg^−1^), and Pb3000 (3000 mg Pb kg^−1^). Data are expressed as mean ± SD (*n* = 10). Due to heteroscedasticity, statistical differences among contamination levels within each species and metal-specific dose series were evaluated using Welch’s ANOVA followed by the Games–Howell post hoc test (*p* < 0.05). Different lowercase letters indicate significant differences among contamination levels within the corresponding species and metal-specific dose series. No statistical comparisons were performed between Cd and Pb treatments.

Cadmium application increased shoot Cd concentrations in both species (Figure 2a). In safflower, shoot Cd reached 12.6 under Cd50 and 277 mg kg^−1^ under Cd100. In chamomile, shoot Cd reached 138.6 (Cd50) and 208 mg kg^−1^ (Cd100). Root Cd concentrations amounted to 42.4 (Cd50) and 305.2 mg kg^−1^ (Cd100) for safflower, and 88.9 (Cd50) and 400.6 mg kg^−1^ (Cd100) for chamomile (Figure 2c). Safflower shoot Pb concentrations reached 66.4 (Pb1500) and 82.3 mg kg^−1^ (Pb3000), with no significant difference between doses. Conversely, chamomile shoot Pb significantly increased from 18.2 (Pb1500) to 92.1 mg kg^−1^ (Pb3000) (Figure 2b). Root Pb concentrations exceeded shoot values in both species reaching 102.3 and 334.1 mg kg^−1^ in safflower and 162.5 and 226.4 mg kg^−1^ in chamomile under Pb1500 and Pb3000, respectively (Figure 2d).

Total shoot Cd and Pb uptake was negligible in controls and non-target metal treatments (Figure 2e,f). In safflower, uptake increased significantly to 30 µg pot^−1^, under Cd50 and peaking at nearly 100 µg pot^−1^ under Cd100 (Figure 2e). In chamomile Cd uptake exceeded 250 µg pot^−1^ under Cd50 and decreased to 50 µg pot^−1^ under Cd100 (Figure 2e). In safflower Pb uptake peaked at 300 µg pot^−1^ under Pb1500 and decreased to 100 µg pot^−1^ under Pb3000 (Figure 2f). In contrast, chamomile shoot Pb uptake was 22 µg pot^−1^ under Pb1500, and dramatically decreased below 6 µg pot^−1^ under Pb3000 treatment (Figure 2f). Expressed relatively to the measured, post-harvest pseudo-total soil metal pool (136.8 mg Cd per pot at Cd50, based on the measured 45.6 mg kg^−1^ in chamomile pots; 3699 mg Pb per pot at Pb1500, based on the measured 1233 mg kg^−1^ in safflower pots; 3 kg soil per pot), this aboveground removal corresponded to approximately 0.18% of the post-harvest soil Cd pool for chamomile at Cd50 (total plant biomass ~1.81 g DW) and approximately 0.008% of the post-harvest soil Pb pool for safflower at Pb1500 (total plant biomass 4.89 g DW), confirming that even the best-performing treatments removed only a minor fraction of the total soil metal content within a single 4-month growth cycle.

To assess whether the chloride accompanying PbCl_2_ contributed independently to the observed growth response, a chloride-only control was included prospectively and conducted concurrently with the main experiment. This treatment received CaCl_2_·2H_2_O at a chloride loading equivalent to that associated with Pb3000 (approximately 1.027 g Cl kg^−1^ soil). No significant differences from the untreated control were detected in shoot DW, root DW, shoot FW, or plant height for either species (Figure S1).

### 3.4. Cd and Pb Mobility in Soil–Plant Interface

**Figure 3:**
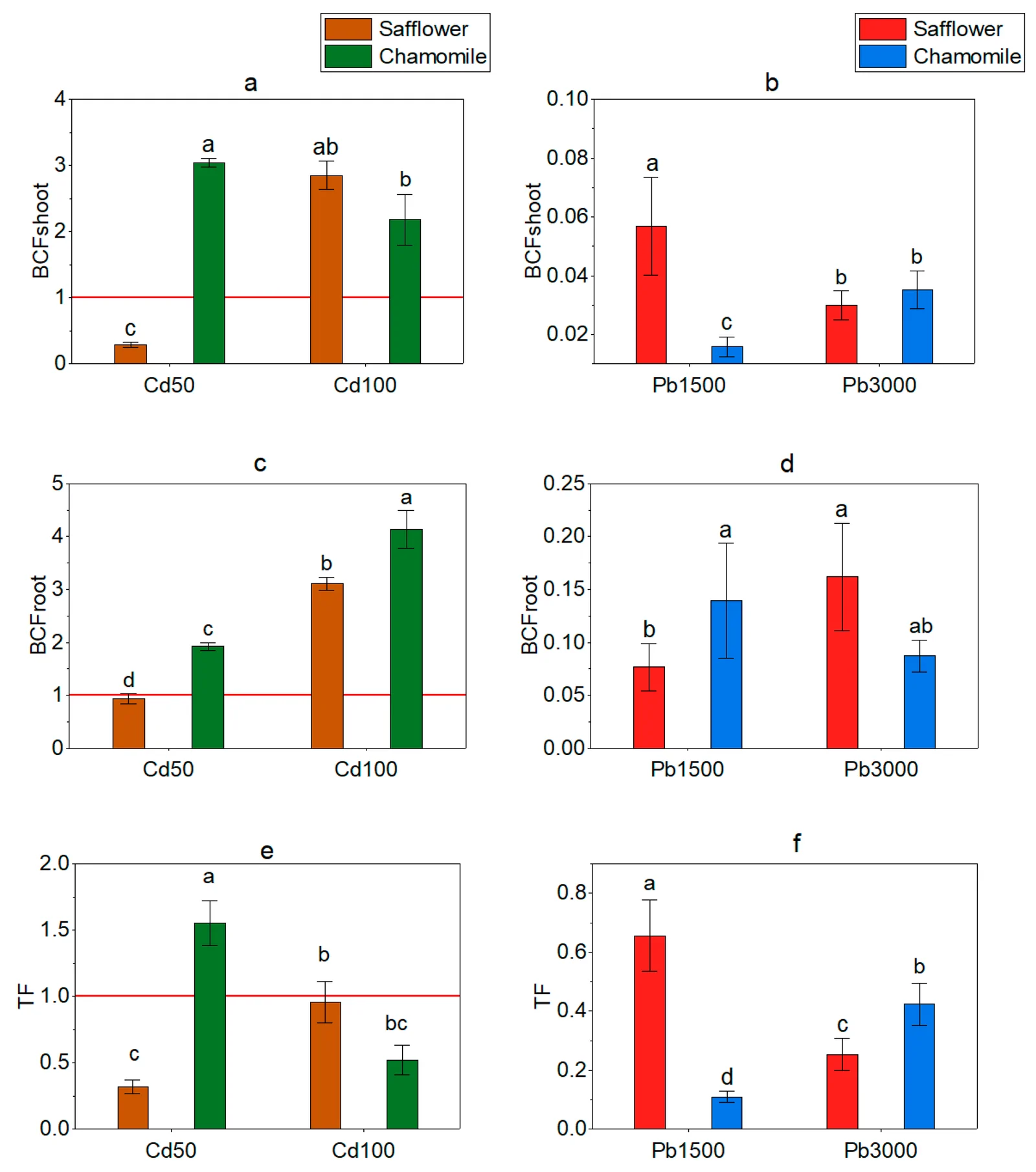
Phytoremediation indices for safflower and chamomile under Cd (left panels: (**a**,**c**,**e**)) and Pb (right panels: (**b**,**d**,**f**)) treatments: (**a**,**b**) shoot Bioconcentration Factor (BCFshoot), (**c**,**d**) root Bioconcentration Factor (BCFroot), and (**e**,**f**) Translocation Factor (TF). The red horizontal line at y = 1 represents the reference threshold used for interpreting accumulation and translocation behavior. BCF and TF values were calculated separately for each experimental unit and are presented as treatment mean ± SD (*n* = 10). Statistical differences among contamination levels within each species and metal-specific dose series were evaluated using one-way ANOVA followed by Tukey’s HSD post hoc test (*p* < 0.05). Different lowercase letters indicate significant differences among contamination levels within the corresponding species and metal-specific dose series. No statistical comparisons were performed between Cd and Pb treatments and between species.

Calculated factors showed species- and element-specific differences across treatments (Figure 3). Regarding BCFshoot, chamomile exceeded unity under Cd exposure (3.04 at Cd50; 2.18 at Cd100), whereas safflower was below unity at Cd50 (0.27) and above unity at Cd100 (2.88) (Figure 3a). Both species exhibited negligible BCFshoot across Pb treatments (Figure 3b). The TF highlighted contrasting internal dynamics; chamomile demonstrated efficient root-to-shoot transfer at Cd50 (1.56), collapsing to 0.52 at Cd100, while safflower kept its TF <1 under Cd50 (0.30) and Cd100 (0.91) (Figure 3e). Under Pb exposure, safflower’s TF decreased from 0.65 at Pb1500 to 0.25 at Pb3000, a trend inverted in chamomile where TF increased from 0.11 (Pb1500) to 0.41 (Pb3000) (Figure 3f). BCFroot was highest under Cd100, reaching 4.20 in chamomile and 3.17 in safflower (Figure 3c). Under Pb treatments, BCFroot remained very low in both species (Figure 3d).

### 3.5. Principal Component Analysis

**Figure 4:**
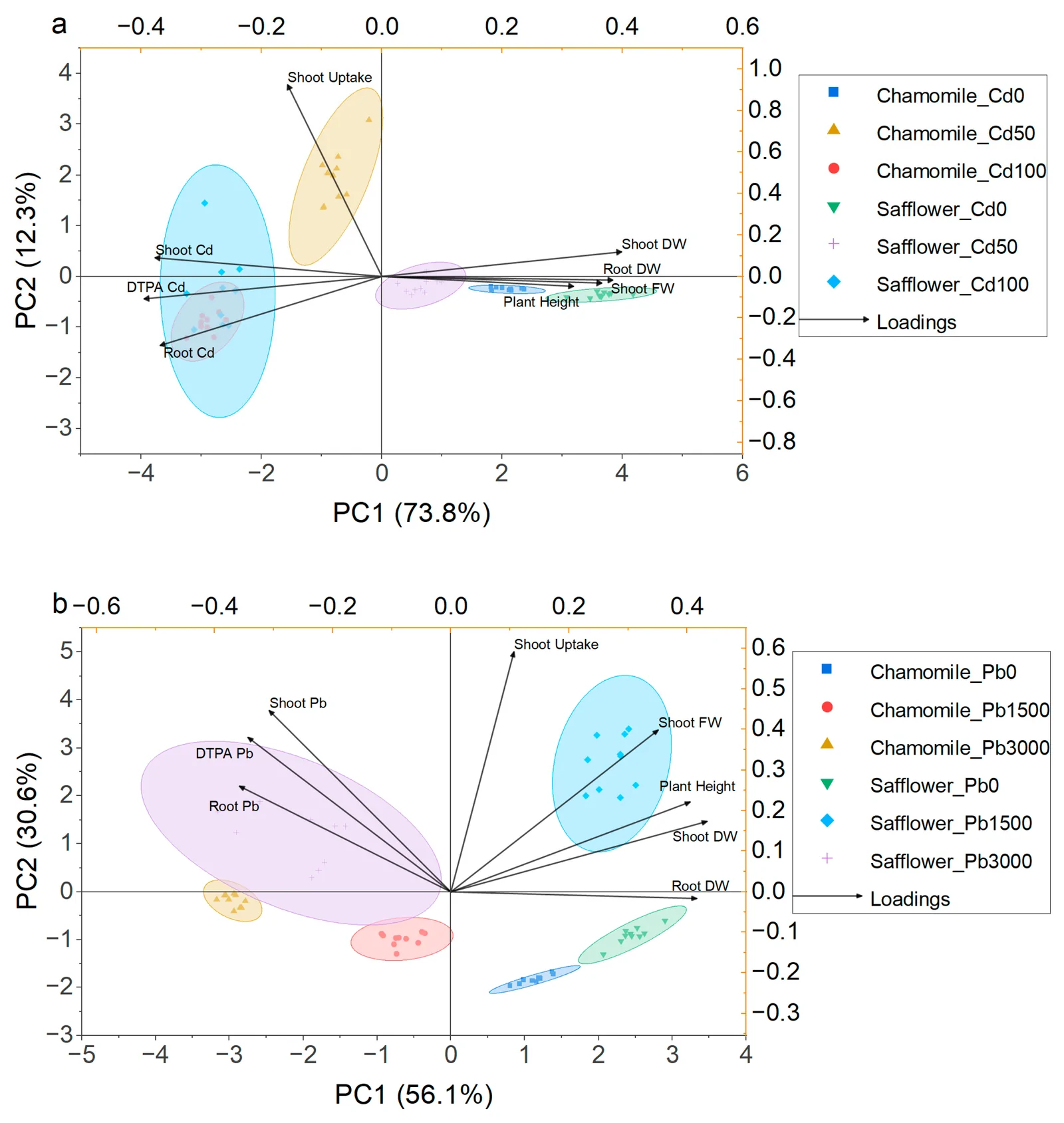
Principal components analysis (PCA) biplots describing multivariate relationships among plant growth, soil metal availability, tissue metal accumulation, and shoot metal uptake in chamomile (*Matricaria recutita* L.) and safflower (*Carthamus tinctorius* L.) under (**a**) Cd and (**b**) Pb exposure. For the Cd dataset, PC1 and PC2 explained 73.8% and 12.3% of the total variance, respectively (86% cumulative variance), whereas for the Pb dataset they explained 56.1% and 30.6%, respectively (86.7% cumulative variance). Variables included shoot DW, root DW, shoot FW, plant height, DTPA-extractable metal concentration, shoot metal concentration, root metal concentration, and shoot metal uptake. Each point represents one independent experimental unit (*n* = 10 per species × treatment group). Ellipses illustrate the distribution of replicate observations within each group. Arrows represent variable loadings; the lower and left axes correspond to PCA scores, whereas the upper and right axes correspond to the loading scale.

Principal Component Analysis was performed separately for the Cd and Pb datasets. The analysis was performed in order to explore multivariate relationships among plant growth parameters, soil metal availability, tissue metal accumulation, and shoot metal uptake (Figure 4). For the Cd dataset, PC1 and PC2 explained 73.8% and 12.3% of the total variance, respectively, accounting together for 86% of the overall variability. Growth-related variables, including shoot DW, root DW, shoot FW, and plant height, loaded positively on PC1, whereas DTPA-extractable Cd and shoot and root Cd concentrations loaded in the opposite direction. Shoot Cd uptake showed its strongest contribution along positive PC2 (Figure 4a). The Cd biplot showed a progressive displacement of the treatment groups away from the controls with increasing Cd exposure. Control plants of both species were positioned on the positive side of PC1, in association with the growth-related variables. The Cd100 treatments were positioned predominantly on the negative side of PC1, closer to the DTPA-extractable and tissue Cd variables. Chamomile at Cd50 was clearly distinguished along positive PC2, in the direction of shoot Cd uptake, whereas safflower at Cd50 occupied an intermediate position between the control and the more strongly Cd-affected groups (Figure 4a).

For the Pb dataset, PC1 and PC2 explained 56.1% and 30.6% of the total variance, respectively, representing 86.7% of the overall variability. Growth-related variables were positioned on the positive side of PC1, whereas DTPA-extractable Pb and shoot and root Pb concentrations loaded in the opposite direction. Shoot Pb uptake was strongly associated with positive PC2, together with shoot FW to a lesser extent (Figure 4b). The score distribution clearly differentiated the response of safflower at Pb1500, which was positioned in the positive PC1–PC2 quadrant in association with shoot uptake and aboveground growth traits. In contrast, Pb3000 shifted both species toward the negative side of PC1, closer to the Pb availability and tissue-accumulation variables. Pb-treated chamomile plants were also clearly displaced from the untreated control along PC1 (Figure 4b).

## 4. Discussion

### 4.1. Plant Growth Under Cd and Pb Stress

Safflower exhibited maximum biomass at Pb1500, with shoot DW and FW increasing by 17% and 44%, respectively, relative to the untreated control. In contrast, biomass declined substantially at Pb3000, demonstrating a clear shift from growth stimulation at Pb1500 to growth inhibition at the higher Pb exposure.

Previous studies have identified several Pb-responsive processes in safflower that may be relevant to this pattern. For example, melatonin-associated mitigation of Pb-induced oxidative stress has been reported in safflower seedlings [20], while Hong et al. [21] demonstrated a role for the Pb-responsive CtWD40-6 gene in Pb uptake and tolerance. These pathways provide plausible physiological hypotheses for safflower responses to Pb stress. However, antioxidant activity, phytohormones, melatonin, gene expression, and related molecular mechanisms were not measured in the present experiment to support this argument. Therefore, our findings should not be interpreted as demonstrated mechanisms underlying the observed Pb1500 growth stimulation.

Conversely, Cd produced markedly stronger growth inhibition at substantially lower nominal soil concentrations than Pb (50–100 mg kg^−1^ Cd versus 1500–3000 mg kg^−1^ Pb) in both species. This stronger phytotoxic response is consistent with the relatively high mobility and efficient plant uptake commonly reported for Cd [44], which can contribute to root injury, disruption of ion homeostasis and inhibition of plant growth [45]. Notably, at Cd50, chamomile height and shoot FW remained unaffected despite a significant reduction in shoot DW. This is aligned with findings that moderate Cd levels do not disrupt certain phenotypic parameters despite decreases in dry biomass [46]. For instance, chamomile exposed to 3–12 mg Cd kg^−1^ maintained unaffected shoot FW, with height reductions typically appearing only at elevated doses [22]. Chamomile’s ability to maintain shoot FW and plant height at Cd50, despite reduced dry biomass, indicates substantial tolerance to Cd exposure under the alkaline soil conditions tested here.

The multivariate analysis further supported these contrasting growth responses. In the Cd PCA, shoot DW, root DW, shoot FW and plant height were associated with positive PC1 scores, whereas DTPA-extractable Cd and shoot and root Cd concentrations were positioned in the opposite direction. This pattern indicates that increasing Cd availability and tissue accumulation coincided with declining plant performance across the tested treatments. A different pattern emerged under Pb exposure: safflower at Pb1500 was positioned in the positive PC1–PC2 region (Figure 4b), in association with growth-related traits and shoot Pb uptake, whereas the highest Pb exposure shifted the observations towards the Pb-related side of the ordination. Thus, the PCA further illustrates the transition from the growth maintenance or stimulation at Pb1500 to pronounced growth inhibition at Pb3000.

### 4.2. Concentrations of Cd and Pb in Plants

#### 4.2.1. Chamomile

Under severe Cd contamination, chamomile accumulated 138.6 and 208 mg Cd kg^−1^ DW in shoots at Cd50 and Cd100, respectively, demonstrating substantial aboveground Cd accumulation. BCFshoot values exceeded unity at both Cd treatments, reaching 3.04 at Cd50 and 2.18 at Cd100. However, internal Cd partitioning changed markedly with increasing exposure: TF was 1.56 at Cd50, indicating preferential root-to-shoot translocation, but decreased to 0.52 at Cd100 as root Cd concentrations increased and plant biomass declined. At Cd50, chamomile maintained shoot FW and plant height relative to the control despite a significant reduction in shoot dry biomass, whereas Cd100 caused severe biomass suppression. Together, these findings indicate strong Cd accumulation capacity but also a clear exposure-dependent limitation in tolerance and shoot translocation. The elevated shoot Cd concentrations observed here are consistent with previous studies reporting substantial Cd uptake and aboveground accumulation in chamomile under hydroponic and soil conditions [13,14].

Our results indicate that chamomile may strongly express its Cd accumulation and translocation capacity under extreme contamination (50 and 100 mg kg^−1^) in alkaline environments. This behavior—maintaining BCFshoot > 1 at both Cd treatments and TF > 1 (only at Cd50)—indicates strong Cd accumulation, while also demonstrating a shift toward greater root retention as Cd exposure increases, a phenomenon that has received limited attention in previous reports in the literature. This accumulation pattern resulted in the highest total shoot Cd removal observed in the experiment, extracting over 250 µg pot^−1^. Nonetheless, a high BCF is not synonymous with substantial bulk soil decontamination: the corresponding shoot uptake represents only a minor fraction of the total applied Cd, indicating that meaningful soil-level Cd reduction would likely require multiple sequential harvests or extended cropping cycles rather than a single growth period. Furthermore, the high accumulation at Cd50 contrasted with the suppressed translocation and biomass at Cd100, being consistent with data showing tolerance limits under severe stress [22]. Previous reports have described substantial variation in Cd partitioning in chamomile, ranging from preferential root retention to substantial aboveground accumulation [13,14,15]. The present results extend this evidence by showing that strong Cd accumulation can occur in a calcareous alkaline soil, while also demonstrating that root-to-shoot translocation declines as exposure and toxicity increase. This exposure-dependent response was also evident in the multivariate analysis. Chamomile at Cd50 was distinguished primarily along positive PC2, in the direction associated with shoot Cd uptake, whereas the Cd100 observations were positioned more strongly toward negative PC1, together with DTPA-extractable and tissue Cd concentrations. This pattern helps explain the contrasting phytoremediation-related responses at the two Cd levels. At Cd50, substantial Cd accumulation occurred while sufficient biomass was retained to produce the highest aboveground Cd uptake, whereas at Cd100 the increase in tissue Cd concentration coincided with severe biomass suppression. Consequently, the higher tissue Cd concentrations observed at the greatest exposure did not translate into greater aboveground Cd removal (Figure 4a). Overall, these findings demonstrate that marked Cd accumulation and dose-dependent changes in translocation can occur in chamomile even under the geochemical constraints of a calcareous alkaline soil. The present design, however, does not allow the specific contribution of soil alkalinity to these responses to be separated from other soil- and exposure-related factors.

At Pb1500, chamomile accumulated substantially more Pb in roots (162.5 mg kg^−1^) than in shoots (18.2 mg kg^−1^), indicating strong belowground retention and limited root-to-shoot translocation (Figure 2b,d). Such partitioning is consistent with root-retention processes described for Pb-exposed plants, including cell-wall binding and intracellular sequestration [36,37]. However, these mechanisms were not directly measured in the present study. Increasing Pb exposure was accompanied by severe reductions in both root and shoot biomass, indicating pronounced toxicity at the highest Pb treatment. Although oxidative stress, disruption of antioxidant systems, and impaired cellular function have been reported under severe Pb exposure in other species [47,48], these physiological responses were not quantified here and are therefore considered possible explanations rather than experimentally demonstrated mechanisms. Consistent with these responses, Pb-exposed chamomile observations were displaced toward negative PC1 scores in the Pb PCA, away from the growth-related variables and toward the DTPA-extractable and tissue Pb variables (Figure 4b).

#### 4.2.2. Safflower

Unlike chamomile, safflower showed predominantly belowground Pb retention, with TF remaining below unity across both Pb treatments. Although the calcareous alkaline soil matrix would generally be expected to favor Pb sorption and precipitation reactions, a substantial DTPA-extractable fraction remained in the present experiment, accounting for approximately 44–47% of pseudo-total Pb at Pb1500 and 51–52% at Pb3000. Thus, the alkaline soil did not prevent the presence of a sizeable chelator-accessible Pb pool, particularly at the highest loading. The increasing DTPA-extractable fraction with increasing Pb addition may reflect progressive saturation of high-affinity retention sites, although Pb speciation was not directly determined in this study.

Despite this measurable soil Pb availability, safflower maintained relatively limited shoot translocation, particularly at Pb3000. This pattern indicates that the low shoot transfer observed here was associated primarily with plant-level partitioning rather than complete geochemical immobilization of Pb in the soil. The growth stimulation observed at Pb1500 may therefore reflect a species-specific physiological response to Pb exposure under the conditions tested.

Because Pb was supplied as PbCl_2_, accompanying chloride represented a potential experimental confounder—elevated soil chloride is generally reported to induce mild osmotic tension or salinity-related growth suppression in safflower, even in this comparatively salt-tolerant species, rather than growth promotion [49]. This possibility was addressed prospectively through a chloride-only treatment that was included in the original experimental design and conducted concurrently with the main experiment. The CaCl_2_·2H_2_O treatment provided a chloride loading equivalent to that associated with Pb3000 (approximately 1.027 g Cl kg^−1^ soil), representing the highest chloride exposure among the Pb treatments. The absence of significant differences between the chloride-only and untreated controls in shoot DW, root DW, shoot FW, or plant height indicates that chloride alone, at the concentration tested, was not sufficient to reproduce the growth stimulation observed in safflower at Pb1500. Nevertheless, potential interactions between chloride, Pb speciation, and plant physiology were not directly investigated and therefore cannot be completely excluded. This result strengthens the interpretation that the observed Pb1500 growth stimulation cannot be explained by chloride exposure alone. Nevertheless, the physiological mechanisms underlying this response were not directly investigated in the present study. Correspondingly, the Pb recovered in safflower shoots at Pb1500 represented only ~0.008% of the measured post-harvest soil Pb pool (~300 µg pot^−1^). Thus, the observed response is more appropriately interpreted as a low-translocation, belowground-retention phenotype compatible with phytostabilization-oriented phytomanagement than as a mechanism for substantial bulk-soil Pb removal. Long-term Pb immobilization, reduced leaching, and the permanence of root-associated retention were not directly assessed. These removal-efficiency estimates are based on the measured, post-harvest pseudo-total soil Cd and Pb concentrations (see Section 2 and Section 3) rather than on the nominal spiked dose, providing a directly measured basis for these calculations. We note, however, that aqua regia digestion yields a pseudo-total rather than an absolute total soil metal content, and that a fully closed mass balance would additionally require complete whole-plant (shoot and root) metal quantification alongside the soil-side measurement; we identify this more complete accounting as a valuable refinement for future work.

The PCA further highlighted the distinct multivariate response of safflower at Pb1500. This treatment was positioned in the positive PC1–PC2 region and was associated with shoot FW, plant height, shoot DW, and shoot Pb uptake. In contrast, safflower at Pb3000 shifted toward negative PC1 scores and toward the DTPA-extractable Pb and tissue Pb variables. The resulting ordination indicates that the response to increasing Pb exposure was not simply monotonic: Pb1500 combined comparatively high biomass with greater shoot uptake, whereas Pb3000 was characterized by increased Pb burden and severe growth inhibition. This multivariate pattern is therefore consistent with a dose-dependent transition from tolerance at the intermediate Pb treatment to toxicity at the highest exposure (Figure 4b).

Under Cd50, safflower retained more Cd in roots (42.4 mg kg^−1^) than in shoots (12.6 mg kg^−1^; TF = 0.30), indicating preferential belowground Cd retention. However, shoot DW significantly declined from 3.5 to 2.3 g, and height was stunted to 13 cm against the control (45 cm). This retardation indicates lower tolerance than chamomile. At Cd100, the marked increase in tissue Cd concentrations coincided with severe biomass suppression, indicating limited physiological tolerance under the highest Cd exposure. The Cd PCA was consistent with this response, as safflower shifted progressively away from the control region associated with plant growth and toward the negative PC1 region associated with increasing Cd availability and tissue accumulation.

Overall, the PCA integrated the univariate responses into two contrasting multivariate patterns. In the Cd dataset, increasing metal availability and tissue accumulation were inversely associated with plant growth, while chamomile at Cd50 retained sufficient biomass to combine substantial shoot accumulation with the highest aboveground Cd uptake. In the Pb dataset, safflower at Pb1500 was associated with growth and shoot uptake traits, whereas the highest Pb exposure shifted the response toward greater Pb burden and reduced plant performance. These multivariate associations reinforce the species- and dose-dependent responses identified by the individual growth, accumulation, BCF, and TF analyses. However, because BCF and TF were evaluated separately and were not included as active PCA variables, the PCA should be considered complementary to, rather than a replacement for, the specific interpretation of metal accumulation and translocation indices. Moreover, PCA describes covariation among the measured variables and does not establish causal physiological mechanisms.

### 4.3. Practical Implications for Single-Metal Contaminated Sites

It should be emphasized that the present findings derive from a short-term (4-month) greenhouse pot experiment, which captures plant tolerance, accumulation, and stabilization responses under controlled conditions but cannot account for long-term soil cleanup rates, field-scale removal efficiency, seasonal and climatic variability, microbial and leaching dynamics, contamination heterogeneity, or biomass disposal logistics. The following considerations are therefore presented as conceptual, bioeconomically oriented directions rather than validated field recommendations. While industrial and historical mining sites often feature multi-metal contamination, evaluating single-metal dynamics remains highly relevant for specific, prevalent real-world scenarios. In Cd-dominated contaminated soils, the high shoot accumulation observed in chamomile suggests potential value as a screening candidate for repeated-harvest phytoextraction-oriented systems. However, the present experiment showed that even the best-performing chamomile treatment removed only approximately 0.18% of the measured post-harvest soil Cd pool during one 4-month growth cycle. Consequently, meaningful soil-level Cd depletion would require evaluation over multiple growth and harvest cycles.

Importantly, the high Cd concentrations measured in chamomile shoots mean that biomass produced on contaminated soil should not be assumed suitable for medicinal, food, or feed uses. Any practical phytomanagement strategy would require contaminant-safe biomass handling, disposal, or an appropriately controlled non-food valorization pathway. The present study did not assess biomass-processing options, regulatory compliance, or economic feasibility; therefore, no conclusions regarding commercial profitability can be drawn. A more immediately applicable phytomanagement context concerns Pb-contaminated sites where safflower’s root-retention pattern is directly relevant—most notably shooting ranges, where alkaline, calcareous soils are common and Pb contamination arises from a well-characterized weathering pathway. In such alkaline environments, discarded metallic ammunition undergoes long-term weathering, oxidizing into litharge (PbO) and eventually converting into thermodynamically stable cerussite (PbCO_3_) via intermediate hydrocerussite Pb_3_(CO_3_)^2^(OH)^2^, driven by dissolved CO_2_ from microbial activity and root respiration [50]. Field studies at abandoned shooting ranges, e.g., in Galicia, Spain [50], and Kaisariani, Greece [51], report total Pb concentrations ranging from 720 to 4800 mg kg^−1^. For such degraded, Pb-dominated sites, cultivating a highly tolerant crop like safflower represents a promising phytomanagement strategy: its predominant root-level Pb retention (TF < 1) is consistent with a phytostabilization-type response limiting shoot-level Pb transfer.

## 5. Conclusions

Our study evaluated the responses of chamomile and safflower to severe Cd and Pb contamination in a calcareous alkaline soil. Chamomile showed high Cd accumulation in shoots at both Cd treatments, with preferential shoot translocation at Cd50 but increased root retention and severe biomass suppression at Cd100. Safflower displayed significant growth stimulation at Pb1500 together with predominantly belowground Pb retention, whereas substantial growth inhibition occurred at Pb3000.

These responses identify potentially useful phytoremediation-related traits rather than demonstrating effective soil remediation. In particular, single-cycle aboveground removal represented only a small fraction of the measured soil metal pools, and the low Pb translocation observed in safflower indicates a phenotype compatible with phytostabilization-oriented management but does not establish long-term Pb immobilization or reduced environmental mobility.

Based on the present findings, future research on chamomile should prioritize repeated-harvest evaluation of its Cd phytoextraction potential at contamination levels where sufficient biomass can still be maintained, whereas safflower should be further investigated for phytostabilization-oriented management of Pb-contaminated soils due to its predominantly belowground Pb retention. Field-scale and multi-season experiments are required to determine long-term metal removal or retention, changes in metal mobility and leaching, responses under heterogeneous contamination, and the influence of soil and environmental variability. Future studies should also address the safe handling or potential non-food utilization of contaminated biomass and evaluate whole-plant metal mass balances before either species can be considered for operational phytomanagement.

## Data Availability

The data that support the findings of this study are available from the corresponding author upon reasonable request due to privacy.

## Supplementary Materials

The following supporting information can be downloaded at: https://www.mdpi.com/article/10.3390/plants15203088/s1, Figure S1: Effect of chloride loading on growth parameters of safflower and chamomile plants; Figure S2: Schematic experimental design of the pot experiment; Table S1: Pseudo-total and DTPA-extractable Cd and Pb concentrations (mg kg^−1^) in soils where safflower and chamomile were grown; Table S2: Summary of statistical test outcomes for all analyzed growth, tissue concentration, uptake, and phytoremediation index variables.
