# Supplementary material for: Growth, Heavy Metal Accumulation, and Translocation in Chamomile (Matricaria recutita L.) and Safflower (Carthamus tinctorius L.) Under Severe Cd and Pb Contamination in an Alkaline Soil
Source: Plants (Basel). 2026 Oct 9;15(20):3088. doi: 10.3390/plants15203088 (PMC13709005; doi:10.3390/plants15203088)
Supplement: Supplementary file 1 [file plants-15-03088-s001.zip › plants-4576719-supplementary.pdf]

Growth, Heavy Metal Accumulation, and Translocation in Chamomile (*Matricaria recutita* L.) and Safflower (*Carthamus tinctorius* L.) Under Severe Cd and Pb Contamination in an Alkaline Soil

Christos Kikis, Aspasia Grammenou, Georgios Thalassinos, Spyridon A. Petropoulos * and Vasileios Antoniadis

Department of Agriculture, Crop Production and Rural Environment, University of Thessaly, 38446 Volos, Greece; chkikis@uth.gr (C.K.); asgrammenou@uth.gr (A.G.); gthalassinos@uth.gr (G.T.); antoniadis@uth.gr (V.A.)

* Correspondence: spetropoulos@uth.gr

Supplemental Material

Table S1. Pseudo-total and DTPA-extractable Cd and Pb concentrations (mg kg^{-1}) in soils where safflower and chamomile were grown.

Plant species	Treatment	Total Cd (mg kg^{-1})	DTPA Cd (mg kg^{-1})	Total Pb (mg kg^{-1})	DTPA Pb (mg kg^{-1})
**Safflower**	Control	0.054 ± 0.02^{a}	0.02 ± 0.01^{a}	3.37 ± 1.16^{a}	0.85 ± 0.46^{a}
	Cd50	46.10 ± 2.33^{b}	13.7 ± 1.37^{b}	3.84 ± 1.01^{a}	1.51 ± 0.72^{a}
	Cd100	96.27 ± 1.91^{c}	51.8 ± 4.81^{c}	3.65 ± 1.01^{a}	1.80 ± 0.50^{a}
	Pb1500	0.02 ± 0.01^{a}	0.01 ± 0.01^{a}	1233 ± 142^{b}	580 ± 83^{b}
	Pb3000	0.04 ± 0.02^{a}	0.01 ± 0.01^{a}	2773 ± 123^{c}	1415 ± 142^{c}
	Significance	$p < 0.001$	$p < 0.001$	$p < 0.001$	$p < 0.001$
**Chamomile**	Control	0.08 ± 0.03^{a}	0.02 ± 0.01^{a}	4.47 ± 1.94^{a}	1.58 ± 0.49^{a}
	Cd50	45.6 ± 2.84^{b}	16.2 ± 2.10^{b}	3.63 ± 1.23^{a}	1.11 ± 0.11^{a}
	Cd100	95.4 ± 1.56^{c}	51.1 ± 6.95^{c}	4.42 ± 1.39^{a}	2.80 ± 0.55^{a}
	Pb1500	0.03 ± 0.01^{a}	0.01 ± 0.01^{a}	1226 ± 164^{b}	542 ± 21^{b}
	Pb3000	0.04 ± 0.01^{a}	0.01 ± 0.01^{a}	2722 ± 219^{c}	1437 ± 124^{c}
	Significance	$p < 0.001$	$p < 0.001$	$p < 0.001$	$p < 0.001$

Treatments included control (unamended soil), Cd50 (Cd added at 50 mg kg^{-1}), Cd100 (Cd added at 100 mg kg^{-1}), Pb1500 (Pb added at 1500 mg kg^{-1}), and Pb3000 (Pb added at 3000 mg kg^{-1}). Values represent means ± standard error ($n = 10$). Different letters indicate significant differences among treatments according to Welch's ANOVA followed by the Games-Howell post-hoc test at $p < 0.05$ due to unequal variances.

Figure S1. Effect of chloride loading on growth parameters of safflower and chamomile plants.

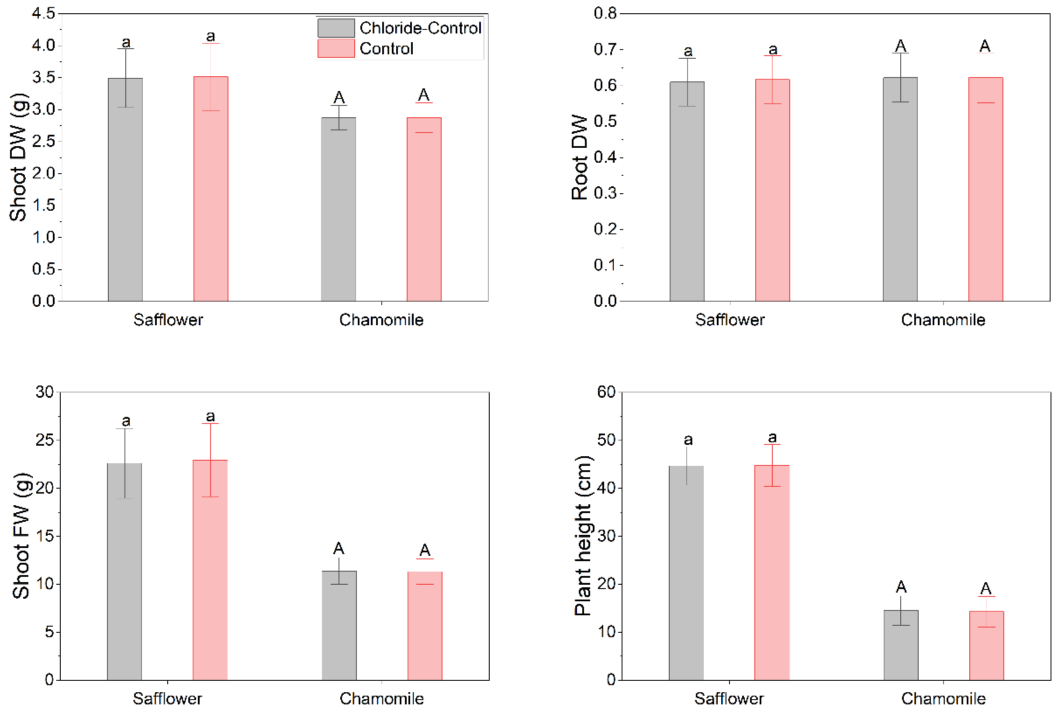

Figure S1. Effect of a chloride-only control on growth parameters of safflower and chamomile plants: (a) shoot dry weight (DW), (b) root DW, (c) shoot fresh weight (FW), and (d) plant height. The Chloride-Control treatment consisted of soil amended with $CaCl_2$ at a chloride loading equivalent to that introduced by $PbCl_2$ at Pb3000 (~1.027 g Cl kg^{-1} soil), without any accompanying Cd or Pb addition, compared against the untreated Control. Data are expressed as mean ± SD (n = 10). Statistical differences were evaluated by one-way ANOVA followed by Tukey's HSD post-hoc test ($p < 0.05$). Different letters indicate significant differences between treatments within each species (lowercase for safflower, uppercase for chamomile); identical letters indicate no significant difference.

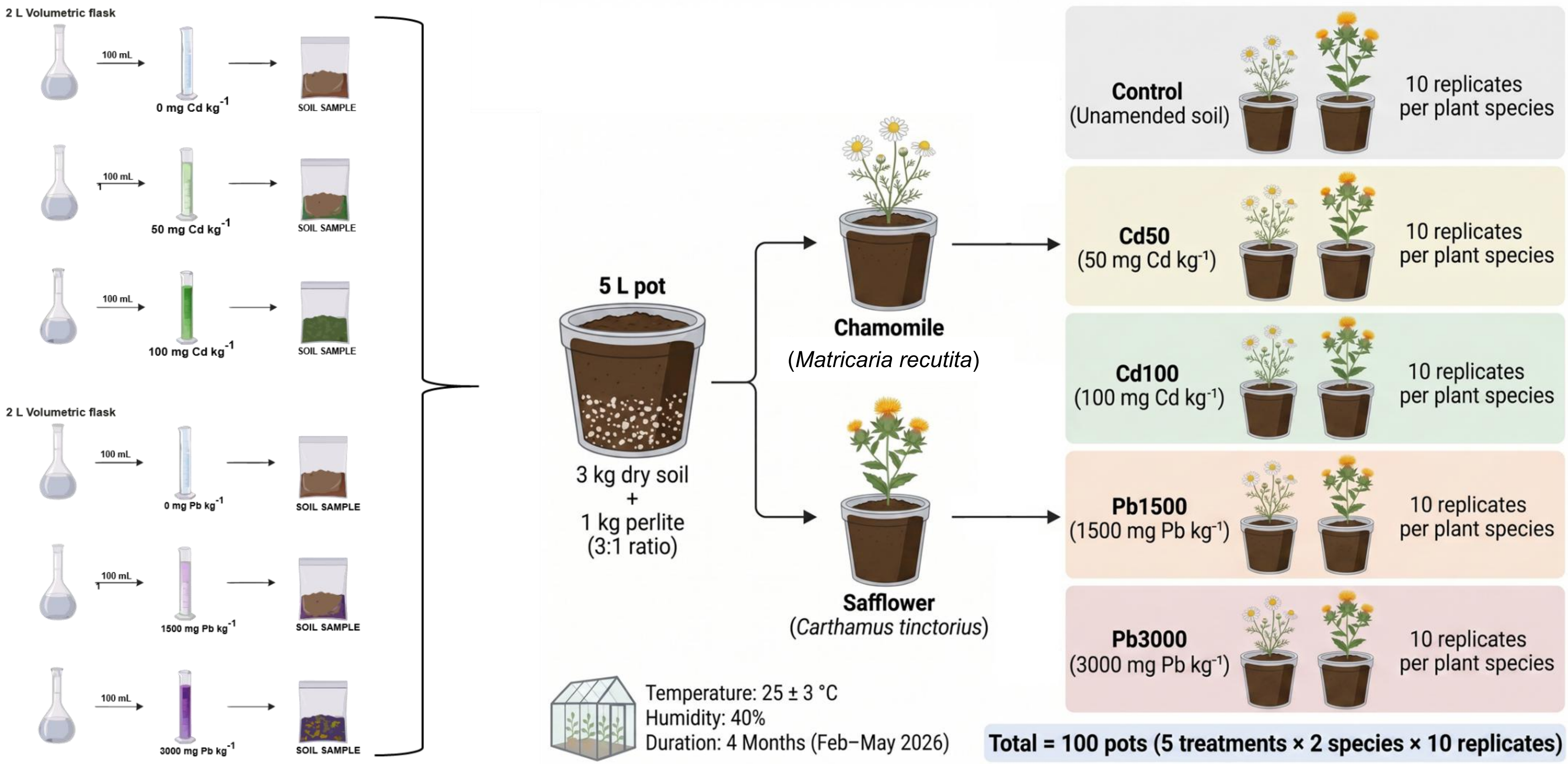

Figure S2. Schematic experimental design of the pot experiment. The workflow illustrates the preparation of spiking solutions (0, 50, and 100 mg Cd kg^{-1}; 0, 1500, and 3000 mg Pb kg^{-1}), substrate mixture configuration (3 kg dry soil and 1 kg perlite in a 3:1 ratio per 5 L pot), and the allocation of the five experimental treatments (Control, Cd50, Cd100, Pb1500, and Pb3000) for chamomile (*Matricaria recutita*) and safflower (*Carthamus tinctorius*) with 10 replicates per plant species (n = 10) total pots) under controlled greenhouse conditions.

Table S2. Summary of statistical test outcomes for all analyzed growth, tissue concentration, uptake, and phytoremediation index variables.

Plant species	Parameter	Treatment factor	Sample size	Statistical Test	dF	F-value	p-value (effect size)
Safflower/Chamomile	Shoot DW	Cd (0, 50, 100 ppm)	10	One-Way ANOVA (Tukey HSD)	5.54	160.83	$p < 0.001$ ($\eta^2 = 0.937$)
Safflower/Chamomile	Shoot DW	Pb (0, 1500, 3000 ppm)	10	One-Way ANOVA (Tukey HSD)	5.54	153.45	$p < 0.001$ ($\eta^2 = 0.935$)
Safflower/Chamomile	Root DW	Cd (0, 50, 100 ppm)	10	One-Way ANOVA (Tukey HSD)	5.54	204.80	$p < 0.001$ ($\eta^2 = 0.95$)
Safflower/Chamomile	Root DW	Pb (0, 1500, 3000 ppm)	10	One-Way ANOVA (Tukey HSD)	5.54	199.33	$p < 0.001$ ($\eta^2 = 0.952$)
Safflower/Chamomile	Shoot FW	Cd (0, 50, 100 ppm)	10	One-Way ANOVA (Tukey HSD)	5.54	102.848	$p < 0.001$ ($\eta^2 = 0.905$)
Safflower/Chamomile	Shoot FW	Pb (0, 1500, 3000 ppm)	10	One-Way ANOVA (Tukey HSD)	5.54	97.338	$p < 0.001$ ($\eta^2 = 0.91$)
Safflower/Chamomile	Plant height	Cd (0, 50, 100 ppm)	10	One-Way ANOVA (Tukey HSD)	5.54	209.499	$p < 0.001$ ($\eta^2 = 0.951$)
Safflower/Chamomile	Plant height	Pb (0, 1500, 3000 ppm)	10	One-Way ANOVA (Tukey HSD)	5.54	188.32	$p < 0.001$ ($\eta^2 = 0.963$)
Safflower/Chamomile	Shoot Cd conc.	Cd (0, 50, 100 ppm)	10	One-Way ANOVA (Games-Howell)	5.54	213.32	$p < 0.001$ ($\eta^2 = 0.953$)
Safflower/Chamomile	Shoot Pb conc.	Pb (0, 1500, 3000 ppm)	10	One-Way ANOVA (Games-Howell)	5.54	179.93	$p < 0.001$ ($\eta^2 = 0.946$)
Safflower/Chamomile	Root Cd conc.	Cd (0, 50, 100 ppm)	10	One-Way ANOVA (Games-Howell)	5.54	247.73	$p < 0.001$ ($\eta^2 = 0.958$)
Safflower/Chamomile	Root Pb conc.	Pb (0, 1500, 3000 ppm)	10	One-Way ANOVA (Games-Howell)	5.54	221.24	$p < 0.001$ ($\eta^2 = 0.952$)

Safflower/Chamomile	Shoot Cd uptake	Cd (0, 50, 100 ppm)	10	One-Way ANOVA (Games-Howell)	5.54	178.88	$p < 0.001$ ($\eta^2 = 0.932$)
Safflower/Chamomile	Shoot Pb uptkae	Pb (0, 1500, 3000 ppm)	10	One-Way ANOVA (Games-Howell)	5.54	182.654	$p < 0.001$ ($\eta^2 = 0.924$)
Safflower/Chamomile	Shoot BCF	Cd (50, 100 ppm)	10	One-Way ANOVA (Tukey HSD)	3.36	186.79	$p < 0.001$ ($\eta^2 = 0.96$)
Safflower/Chamomile	Shoot BCF	Pb (1500, 3000 ppm)	10	One-Way ANOVA (Tukey HSD)	3.36	201.44	$p < 0.001$ ($\eta^2 = 0.968$)
Safflower/Chamomile	Root BCF	Cd (50, 100 ppm)	10	One-Way ANOVA (Tukey HSD)	3.36	184.22	$p < 0.001$ ($\eta^2 = 0.972$)
Safflower/Chamomile	Root BCF	Pb (1500, 3000 ppm)	10	One-Way ANOVA (Tukey HSD)	3.36	174.11	$p < 0.001$ ($\eta^2 = 0.954$)
Safflower/Chamomile	TF	Cd (50, 100 ppm)	10	One-Way ANOVA (Tukey HSD)	3.36	181.91	$p < 0.001$ ($\eta^2 = 0.955$)
Safflower/Chamomile	TF	Pb (1500, 3000 ppm)	10	One-Way ANOVA (Tukey HSD)	3.36	192.33	$p < 0.001$ ($\eta^2 = 0.954$)

For each variable, the applied statistical test (one-way ANOVA followed by Tukey's HSD for homoscedastic data, or Welch's ANOVA followed by Games-Howell post-hoc test for heteroscedastic data), sample size per group (n), degrees of freedom (df), test statistic (F or Welch's F), exact p-value, and effect size (η^2) are reported. Cd and Pb treatments were analyzed as separate experiments for each species
